# Implementing a one health approach to strengthen the management of zoonoses in Ethiopia

**DOI:** 10.1016/j.onehlt.2023.100521

**Published:** 2023-03-03

**Authors:** Ndungu S. Nyokabi, Henrietta Moore, Stefan Berg, Johanna Lindahl, Lisette Phelan, Gizachew Gimechu, Adane Mihret, James L N Wood

**Affiliations:** a Institute for Global Prosperity, University College London, United Kingdom; b Bernhard Nocht Institute for Tropical Medicine, Hamburg, Germany; c International Livestock Research Institute (ILRI), P.O. Box 30709, Nairobi 00100, Kenya; d Department of Medical Biochemistry and Microbiology, Uppsala University, P.O. Box 582, 75123 Uppsala, Sweden; e Department of Clinical Sciences, Swedish University of Agricultural Sciences, P.O. Box 7054, 75007 Uppsala, Sweden; f School of Geography, University of Leeds, United Kingdom; g Armauer Hansen Research Institute (AHRI), Ethiopia; h Department of Veterinary Medicine, University of Cambridge, United Kingdom

**Keywords:** One health, One health framework, Zoonoses, Integrated service delivery, Community health workers, Health practitioners, Infection prevention and control, Multidisciplinary research

## Abstract

In East Africa, a region with many endemic and emerging zoonoses, and in countries such as Ethiopia in particular, One Health (OH) approaches are increasingly seen as effective ways, to mitigate the risk of zoonoses at the interface between human, animal and the environment. The OH approach promotes interdisciplinary cooperation and collaboration between researchers and practitioners from the disciplines of human, animal and environmental health. Moreover, it advocates for the establishment of a public health sector model which recognises the imperative to holistically address diseases that occur in the human, animal and environmental health arena.

Key informant interviews were conducted with human and animal health practitioners and academic researchers in Ethiopia to collect data on the implementation of the OH approach to manage zoonotic diseases at the human and animal health interface. Participants' observations were undertaken within animal and human health clinics and government laboratories to gather additional data. Environmental health was not considered in this study as it is not yet fully integrated into the OH approach in Ethiopia.

The results reveal a lack of interdisciplinary cooperation, collaboration, and coordination between animal and human health practitioners in operationalising the OH framework in Ethiopia. Professionals in academic and non-academic institutions and organisations are interested in implementing the OH approach, however, an organisational “silo” culture constrains collaboration between institutions dealing with animal and human health. Understaffing and underfunding of institutions were also cited as major challenges to the implementation of a OH approach. Lack of interdisciplinary training for animal and human health practitioners hinders collaboration in the management of zoonoses.

Policymakers need to go beyond the rhetoric to a genuine focus on reform of health management and implement policies that bridge human, animal and environmental health. There is a need for multidisciplinary and transdisciplinary training in human, animal and environmental health and collaborative research for the management of zoonoses.

## 1 Introduction

‘One Health’ (OH) is a holistic interdisciplinary approach to managing public health that conceptualises human, animal and ecological health challenges as interconnected and calls for collaboration at the interface of human, animal, and environmental health [5,18]. It is an approach which aims to break down sectoral “silos” and create a more integrated approach for research, surveillance and response to emerging and endemic infections, involving medical science, public health, veterinary science, ecology, conservation biology and social science [3,5]. Globally, countries are developing their OH frameworks and strategies for implementation in a bid to expand interdisciplinary collaboration and communications regarding all aspects of health care for humans, animals and the environment [3,5,27]. This shift in policy-making and implementation is informed by the realisation that there has been little research on the interaction between human, animal and ecosystem health and that their intersection is poorly understood even though it lies at the heart of public health [5]. The majority of countries globally have traditional disciplinary specialisms, divided policy efforts and compartmentalised funding flow for specific sectoral mandates [5].

Zoonotic diseases pose a threat to human and animal health resulting in illness, loss of productivity and death [22] and have a negative societal impact as they undermine the livelihoods of people who depend on livestock production and/or participate in the trade of animal products as a source of income [4]. Most emerging infectious diseases which affect humans originate from animals [5,22]. Growing recognition that many animal diseases are potentially zoonotic diseases that can cause human illness, death, and economic and societal loss has led to calls for policy-making that pays attention to why zoonotic diseases emerge and how they affect different groups of people in society [3,5].

Policymakers often take a narrow view of public health, emphasising aspects that affect humans but failing to consider non-human factors that significantly contribute to the emergence and threat posed by zoonoses to society [18,22]. Policymaking and evidence-based decision making related to the management of zoonotic diseases, which is underpinned by a OH approach to addressing public health challenges can provide a more complete picture of the opportunities for outbreak prevention than a traditional one-dimensional “siloed” approach [16]. Studies have suggested the need for multi-sectoral collaborations to strengthen disease surveillance systems in humans and animals [22]. Furthermore, studies have indicated there is a need to develop and enhance laboratory capacity and investment in disease prevention and control strategies to mitigate human and animal health risks in global south [22]. Such findings have been translated into policies in some countries in East Africa. For example, Tanzania has a OH platform established by law and placed under the office of the Prime Minister giving it a high decision-making power [15]. Kenya has created a OH-directed Zoonotic Disease Unit (ZDU) which is tasked with coordinating responses to zoonoses outbreaks [14,22]; another example is the Coordinating Office for the Control of Trypanosomiasis in Uganda (COCTU) [25]. Other countries have continued to establish ad-hoc technical committees to manage outbreaks or urgent threats, with these committees having a limited lifespan and scope, and being disbanded once the outbreak has been contained or reduced [7,20].

Ethiopia has the largest livestock population and the second largest human population in Africa [7,22]. Agriculture is important for the country's economy and supports approximately 80% of the population that is reliant on livestock production for its livelihood [4,22]. There is a high prevalence of zoonotic diseases in Ethiopia, including rabies, echinococcosis, brucellosis, bovine tuberculosis, Rift Valley fever (RVF), and Q-fever among others [4,6,10]. The adoption of a OH approach is important as the majority of the Ethiopian population is vulnerable to the risk of zoonotic diseases due to regular direct contact with livestock, consumption of animal source foods (e.g., drinking raw milk or raw blood, or consumption of raw meat), inadequate access to medical treatment and facilities, and living in challenging environmental conditions shared with livestock [4,7,10].

The Ethiopian government has shown strong political commitment to improving the capacity of ministries to address identified gaps in communication and collaboration in the realms of human, animal, and environmental health [21]. Over the last two decades, Ethiopia has established a number of OH initiatives as part of its Global Health Security Agenda (GHSA) commitments to prevent, detect, and respond to existing and emerging threats [7,20,21]. Its GHSA has a OH framework and activities for improving global health security [22]. Ethiopia has established a National One Health Steering Committee (NOHSC) and a number of zoonoses technical working groups (TWG), and has been implementing a five-year OH strategic plan for the period from 2018 to 2022 [20,22]. In addition there are universities that have established one health platforms and training curriculum for training health professionals [21]. There has been progress in Ethiopia against this plan, including the extension of an OH scheme to the regional governments, joint vaccination activities against zoonotic diseases, prioritisation of zoonotic diseases, joint disease surveillance and outbreak investigation activities, development of control and prevention strategies documents for different prioritised zoonotic diseases as well as organising OH and world rabies days celebration [7,20]. There has been limited monitoring and evaluation of OH activities to identify success stories and areas for improvement [20]. Moreover, there is limited awareness regarding the OH principles and their importance, both at a community level and among responsible public sector bodies [7].

Although research on OH has been conducted extensively across the world, only a handful of studies have focused on Ethiopia. The country continues to rely on ad-hoc cross-sectoral committees and initiatives with limited lifespans and scopes to manage zoonoses risk [21]. Policymakers, together with academic and non-academic institutions in Ethiopia, have developed an OH framework, however, the extent of its implementation has not been well investigated, despite Ethiopia continuing to face public health challenges requiring a multidisciplinary and multisectoral response. Taking a qualitative inductive inquiry approach, this study explores the extent of OH implementation in Ethiopia and the impact of such an approach on human, animal, and environmental health.

## 2 Methodology

### 2.1 Study location

This study was undertaken in the Addis Ababa and Oromia regions of Ethiopia. These regions have a high population of consumers of animal source foods living and working in the rapidly growing city of Addis Ababa, the capital city of Ethiopia [6]. The region is comprised of urban and peri-urban areas and is undergoing unplanned rapid urbanization, which creates an animal-human-environment interface that favours disease spill-overs [2,6]. Unplanned urbanization creates health challenges such as absence or insufficient hygiene infrastructure (i.e., lack of sewer lines), peri-urban poverty, low access to health services and antimicrobial resistance [1,12,13]. The predominant livestock-based economy in the region supplies milk and meat products for the urban markets [6,17]. The region hosts slaughterhouses, milk processing companies, butcheries and eateries, supermarkets and informal retailing shops [6].

The research had ethical clearance from the University College London Research Ethics Committee (UCL-REC) approval number 19867/001 and the Armauer Hansen Research Institute (AHRI) and ALERT hospital AHRI/ALERT Ethics Review Committee (AAERC) approval (Protocol number PO-(46/14).

### 2.2 Participant selection

This study involved semi-structured key informant interviews with key stakeholders in human, animal and environmental health institutions in Addis Ababa and Oromia regions. The actors included three laboratory diagnostic researchers from the Animal Health Institute (AHI) - formerly known as the National Animal Health Diagnostic and Investigation Centre (NAHDIC); two researchers from the Ethiopia Public Health Institute (EPHI); six medical doctors (working in Addis Ababa city); one researcher from the Ministry of Agriculture (MoA); one researcher from the Ethiopian Wildlife Conservation Authority (EWCA); one researcher from the Environmental Authority (an agency under the Ministry of Environment, Forest and Climate Change; and 13 veterinary professionals (working in Oromia and Addis Ababa city). Five of the interviewees had previously worked in the NOHSC and/or as experts in disease-specific technical working groups (TWGs) for tackling zoonotic diseases. A number of the researchers were also working as researchers and lecturers in Ethiopian universities.

The participants in this study were chosen through a purposive, snow ball and convenience sampling strategy, and selected based on their (i) experience working in either human, animal and/or environmental health; (ii) willingness to freely participate in the interviews; (iii) experience of working within the study regions. The selected candidates included public health officers, doctors, nurses, community animal health workers, laboratory technologists, policymakers, academicians, and veterinarians, among others. We conducted semi-structured interviews with human health, animal health, and other public health stakeholders, utilizing a grounded theory study design. The semi-structured interviews covered topics such as collaboration, coordination, funding, and staffing of human and animal health services. This study also explored how a rabies control network in Addis Ababa region was being implemented to explore the success stories and possible areas of improvement. Interviews were conducted in English and the local languages of Amharic and Oromo, and were recorded with the prior consent of the participants. Additional data were collected through participant observations undertaken at the human health clinic, animal clinics, a referral hospital and diagnostic laboratories. The data were recorded as notes and pictures with the prior consent of the participants.

The recorded discussions were transcribed and translated by the research team who were familiar with English, and the Oromo and Amharic languages. The transcripts were checked for consistency against the recordings to ensure that meaning was not lost during translation. An inductive approach to thematic analysis was employed to identify key themes regarding the implementation of OH in Ethiopia, as has been described by Griffith et al. [10]. Thematic analysis of the data was undertaken using NVIVO software version 16 (QSR International Pty Ltd. 2018, Chadstone, VIC, Australia).

## 3 Results

### 3.1 Participant characteristics and the provision of human, environmental and animal health

Laboratory professionals worked in specific government laboratories that performed disease diagnostics on either animal or human samples. There were no government laboratories that combined both animal and human sample diagnostics. Interviewed participants highlighted the zoonotic risks faced by the human population and that were driven by close contact between smallholder farmers and their livestock, and by the common practice of consuming raw milk and raw meat:

*“There is a habit of eating raw meat in our area and beef tapeworm* [*Taenia saginata*] *is a common problem”*

**Veterinarian 1**

*“*[a few years ago] *people in our district slaughtered cattle for the annual festival celebration called ‘Kirchea’ and they got sick with anthrax by eating meat, the majority of them were hospitalized and recovered from the disease but some of them died”*

**Veterinarian 2**

Misuse of antibiotics and poor animal waste management practices were also reported. Observations revealed that dead animals and animal manure were often dumped on the side of the roads, or by the river streams or on open fields, actions which increase the risk for environmental contamination that can facilitate disease transmission.

The medical professionals in this study worked in human health centres and clinics in Woredas (district level) and they occasionally visited Kebele(s) (i.e. the smallest administrative unit in Ethiopia) to provide healthcare services. The interviewed medical and veterinary personnel of Addis Ababa perceived the number of health centres and animal clinics as too low to sufficiently serve the large population in the capital, and reported feeling the strain of being overworked:

“*The challenge is the patient load* [..] *we are taking referral patients from other health centres. That is adding a burden on us as well it is delaying the time that patients get treatment. Other health centres in the city should start the service* [for rabies treatment]*, that* [could] *decrease the load from us as well as decrease waiting times for patients*.”

**Medical doctor 1**

Veterinary professionals working in animal clinics located in each Woreda provided services to local farmers at a subsidised price. However, private veterinary professionals were not common, especially in peri-urban and rural areas, due to the associated operational costs, such as transport costs. Moreover, poor farmers were reported as not being able to afford to pay for veterinary services.

*“Farmers bring their cattle when they get sick.* [..] *a professional may go to their place to treat their cattle* [..] *We also provide vaccine services* [..] *prevention is the better option that comes first. We ensure prevention by vaccination and educating the cattle owners.”*

**Veterinarian 3**

In the case of a disease outbreak that affects the human population, the Ethiopia Public Health Institute (EPHI) was reported as the first to be informed and also tasked with responding to contain its spread:

*“When an outbreak is reported to the Ministry of health and agriculture, the EPHI have laboratories that perform laboratory analysis. The researchers of the institutions respond in case of emergency such as a disease outbreak.”*

**Researcher 3, AHI**

*“I have participated in anthrax investigation with public health staff from EPHI. We have done this by communicating with each other.”*

**Researcher 1, AHI**

The Animal Health Institute (AHI) is involved in the sampling and diagnosis of cases of livestock disease outbreaks; however, the responses were not coordinated from a central focal point and thus different institutions were reported as receiving information at different times, which was perceived as leading to a lack of coordinated approach in disease surveillance and surveys:

*“EPHI gets information of diseases outbreak from the local health office and report to us* [diagnostic laboratories] *to investigate the problem, so they have information before us***”**

**Researcher 1, AHI**

The MoA organises an annual surveillance for diseases of priority. Disease prioritisation for an annual surveillance is based on the known socioeconomic impacts of different diseases. These annual surveillances by MoA are however, not coordinated with the Ministry of Health and the Public Health Department, despite that these animal diseases are also zoonotic and are affecting the human population.

“*We plan annual surveillance of zoonotic diseases like bovine tuberculosis, RVF* [Rift Valley fever]*, anthrax* […] *we collect data and samples for analysis* […] w*e consider the economic burden of animal diseases and the public health importance*”

**Researcher 1, AHI**

### 3.2 Implementation of OH framework

Interviewees working for the MoA detailed the process by which Ethiopia had established the NOHSC in 2017. They explained that the steering committee comprised individuals who officially represented the Ministries of Health (MoH**)**, Livestock and Fisheries, Environment, Forest and Climate Change (MoEFCC), Culture and Tourism, Education, Agriculture and Natural Resource (MoA). Additionally, Government institutions either independent or under the various ministries were involved in NOHSC including EWCA (Ethiopian wildlife conservation), Wildlife Authority, Universities, Disaster and Risk Management Commission. The chair and secretary of the NOHSC are rotated every six months. NOHSC works closely with a wide range of international organisations, partners research and development organisations including United States Centre for Disease Control (CDC), Food and Agricultural Organization (FAO), United States Agency for International Development (USAID), World Health Organization (WHO), OHIO Global One Health Initiative, International Livestock Research Institute (ILRI), The One Health for Humans, Environment, Animals and Livelihoods (HEAL) project and international universities. The Ministry of Education. NOHSC memorandum of understanding and terms of reference were developed after a benchmarking exercise based OH frameworks of other countries, such as Uganda and Kenya. The NOHSC of Ethiopia was reported as having established disease-specific technical working groups (TWGs) (e.g., Anthrax Technical Working Group, the National Brucellosis Technical Working Group (NBTWG), the Emerging Pandemic Threats (EPT) technical working group) as well as expert forums (e.g., the National One Health Communication Taskforce (NOHCTF) for tackling the prioritised zoonotic diseases.

“[NOHSC] *does capacity building through the regional and laboratory personnel from public health and animal health* [..] *we have developed strategies for the prioritised zoonotic diseases* [..] *we conduct outbreak investigation activities* [..] *we undertake capacity building by awareness creation* [..] *we also hold international events like one health day, rabies day*”.**Researcher, MoA**

NOHSC facilitated institutionalising OH work at the national level. Additionally, it was reported as overseeing and supporting OH work at regional level by helping the regional governments establish their own OH task forces.

“*We have established task forces in seven regions; Oromia, Amhara, South nation and nationalities, Tigray, Benishangul Gumuz, Gambella, and Somali”*

**Researcher, MoA**

The OH framework was not institutionalized, however, and therefore there was a reliance on ad-hoc TWGs. The absence of institutionalisation meant there was no fixed budget or legal framework and research had to be dependent on donor funding:

“*Due to a lack of institutionalisation, most activities are not done and we face many challenges* [..] *our next plan is to make higher level advocacy* [..] *our aim is to institutionalise One Health and have it in the government organogram*”

**Researcher, MoA**

“*We in EPHI try to suggest the formation of an institute under [the department of] public health which has [an] organogram with* [a] *budget, since the domain of the work is public health even if the diseases arise from the environment and animals*”

**Researcher 1, EPHI**

### 3.3 Disease prioritisation in Ethiopia

Interviewees working for the AHI and EPHI reported that the Ethiopian government had an OH framework that directed government research at the national level and coordinated the response to zoonotic diseases:

*“There is a steering committee at national level and they communicate with each other on specific zoonotic diseases, under them, there is a technical working group and they update each other frequently*.”

**Researcher 2, AHI**

Prioritised diseases included rabies, anthrax, brucellosis, leptospirosis and echinococcus. In 2020, the NOHSC revised the list of diseases and removed echinococcus and leptospirosis from the list and instead included Rift Valley fever (RVF) and pathogenic avian influenza based on a reprioritisation exercise and expert consultation. This reflected the perceived transboundary risks from Kenya and South Sudan, respectively. The NOHSC was reported as having developed strategies for the control of anthrax, rabies, and brucellosis, and as having facilitated the surveillance of disease outbreaks across Ethiopia, including outbreaks of unknown camel disease, wild bird death, rabies and anthrax.

The list of diseases of priority developed by the NOHSC was reported as being influenced by external factors. Although the reprioritisation exercise for prioritised diseases was Ethiopia government-led, there was shared opinion that international donors and bilateral development agencies sometimes influenced national decisions based on their funding priorities. Moreover, global trends such as the rise in antimicrobial resistance (AMR) and pandemics such as Covid-19 also shaped the national research and investment focus.

*“We do cultures of salmonella spp., E. coli, staphylococcus spp*. [from meat and milk], *TB and anthrax in our* laboratory […] *we collect samples from the field.* [...] *we are also doing an anti-microbial susceptibility test to investigate drug resistance* [..] *I have been doing surveillance research on brucellosis* [in a CDC-funded project] *we do activities ourselves and report to the funder* [CDC allocates the resources]*. For example, there is ongoing research on TB funded by different countries that are providing financial resources*”

**Researcher 1, AHI**

“*The one health platform does prioritisation using a CDC tool, which is American experience and the FAO tool, so there is involvement of international organization*. [..] *I cannot say the government* [which is the decision maker] *is not involved in each activity since partners finance activities but the majority of activities are undertaken by the government ministries such as Agriculture, environment and health ministries*”

**Researcher 1, EPHI**

There were divergent views regarding the diseases that should be prioritised, with veterinarians and human health professionals alike expressing their concerns that the government was not prioritising echinococcus and leptospirosis for annual surveillance, despite these zoonoses posing a risk in Ethiopia, and instead replacing these diseases on the priority list with RVF and pathogenic avian influenza. The majority of interviewees felt that rabies, anthrax and brucellosis research and control should be well funded as these zoonoses were causing many deaths and affecting communities, in comparison to RVF and pathogenic avian influenza:

“[There are a lot of diseases that are harming the community] *brucellosis* […] *anthrax* […] *the rabies incidence has increased* [in my view] *rabies is devastating by killing even members of the same family*

**Researcher government lab, 1**

“[I will prioritise] *brucellosis* [..] *anthrax* [..] *then rabies for surveillance”*

**Researcher 3, AHI**

### 3.4 Lack of coordination Institutional culture and communication structure

Interviewees reported that the institutional culture of government departments in Ethiopia was dominated by “silo” or departmental thinking with no collaboration between the human and animal health and environmental departments. Changes in working attitudes and practices were reported as being slow, as were changes in relationships between veterinarians, medical doctors, extension workers, biological scientists, environmental workers and public health workers. Changes in attitudes and relationships were attributed to the provision of training in the aftermath of the Covid-19 pandemic. There was an absence of a OH approach thinking, particularly in the management of food safety, which was alluded to by interviewees:

“*The challenge of collaboration between animal and public health is there is no platform to bring sectors together to discuss on the issue rather than joint emergency responding*. [..] *After we have identified the [disease] causative agent we report in the monthly report to state Minister of Agriculture, we do not have a specific unit to report zoonotic diseases*”.

**Researcher 1, AHI**

Conflict between institutions was reported as being common stemming from competition for resources and disagreements over who should take the lead in implementing the OH strategy. Additionally, collaboration was seen as slowing down due to differences in institutional working culture:

“*The challenges are there are many sectors involved in this work including NGOs. There are many international and multilateral organisations like FAO, WHO and USAID rather than the four key sectors that signed the memorandum of agreement. Others are not interested in having this platform established as an organization* […] *instead they help single activities and are not willing to pool resources* […] *we have created an agenda to mobilize funds from the government, but this is not possible as we do not have an Organogram (formal organisation recognition)*”

**Researcher 1, EPHI**

The main barriers to collaboration highlighted by interviewees were a lack of a coordinating framework and a formal department handling the communication and collaboration between human, animal and environmental health professionals which constrained the response to disease outbreaks. There were efforts to increase collaboration but progress was reported as being slow:

“[Collaboration] *is weak. We had a meeting with EPHI three months ago to plan and work together on antimicrobial resistance of staphylococcus, E. coli and salmonella, but it has not been practical. Work has started for joint integrated national surveillance”*

**Researcher 1, AHI**

*“Most of the focus is only given to human health and most of the time we get outbreak information from EPHI. There is also a passive report to the Ministry of Agriculture without confirmation after an outbreak has already spread”*

**Researcher 2, AHI**

The lack of a central department that collates information and reports from animal and medical clinics slows down a public health response. In the absence of a data management framework, there was a lack of reporting and centralised data management. This reflected the “silo” working culture of the Ethiopian health sector and institutions:

“*Data sharing and management is weak for OH in Ethiopia* […] *it should be mainstreamed in every sector* […] *there is no horizontal collaboration*”

**Researcher 1, EPHI**

Outbreaks of human and livestock diseases were reported through different channels, including by the ministries of health, livestock and fisheries and/or agriculture. The lack of a coordinated reporting channel led to late reporting and underreporting of disease cases, and also hampered response efforts in cases of outbreaks:

*“The reporting has a problem, there is under-reporting and late reporting* [in cases of disease outbreaks] *when we arrive the community would inform us that a disease case happened two weeks ago, this due to long process of the reporting system in which kebele report to district, district to zone, zone to a regional lab, regional lab to the regional agricultural bureau, region report to NAHDIC or Ministry of Agriculture.”*

**Researcher 1, AHI**

Although there was regular weekly reporting of disease by the veterinary and human health clinics, different reporting procedures and communication channels were used and the two departments were cited as not being able to generate centralised information on human or domestic animal diseases, even in cases where diseases were transmitted by a common pathogen.

“*Sometimes when there is a disease outbreak which should be controlled by them* [EPHI] *and there are diseases that have to controlled by our team* [AHI] [….] *often* [there *are* challenges], *public health peoples get information before us, and for example, on anthrax outbreak, they will arrive early and decide to burn the carcass before we* [AHI staff] *do our investigation and collect samples”*

**Researcher 2, AHI**

Similarly, laboratory results were reported through long channels of communication, that constrained rapid responses to disease outbreaks.

“*We formally report to the local agricultural office with recommendations and* [the] *Ministry of Agriculture* [and one member of the steering committee from the Ministry of Agriculture] *communicates the results***Researcher 3 AHI**

The professional interviewees were aware of the importance of collaboration and coordination between their departments and also the importance of taking a OH approach. Additionally, there was a consensus that collaboration, communication and joint surveillance could open up opportunities for collaboration in other matters of public health importance.

*“It is nice for disease investigations and control by collecting data from a different perspective and creating control strategies on both sides[..] The advantage of collaboration is it is good to respond parallel both for animal and public health, otherwise, we do only for animal health alone, so the presence of public health team is advantageous”*

**Researcher 1, AHI**

“*We lack facilities, wildlife veterinarians and capacity for taking samples* [..] *the other benefit* [of one health platform] *is we do not have equipped laboratory, so we undertake diagnosis at NAHDIC* [..] *we have future plans to investigate highly pathogenic avian influenza during* [birds] *migration period on selected spots in Rift Valley lakes with NAHDIC*”

**Researcher, EWCA**

Lack of coordination in cases of joint research work led to dissatisfaction among participating researchers due to perceived different treatment and favouritism based on which research organisations they belonged to:

*“We travelled to field separately and we did not share logistic* [only once did we share logistic] *the budget was coordinated separately for the activities* [not pooled] *But it will be better if we share logistics to reduce the costs and work simultaneously, otherwise, one team arrives at the field site early and they do not care about the other team's activities”*

**Researcher 1, AHI**

The participants reported that there was a low investment in human and animal health services. Clinics and other institutions were understaffed which constrained service delivery. Similarly, veterinarians also opined regarding the understaffing of animal health clinics. Procurement of medical and pharmaceutical products was described as a long bureaucratic process that was undertaken at the end of each budget year. Due to changes in market prices and an increase in demand for animal services, medicines purchased were often not enough to last the whole year and, as public clinics were unable to offer services, farmers had to purchase medicine in private pharmacies.

*“I think* [the budget for our clinic] *is around 300,000 ETB* (1 Ethiopian Birr = 0.019 United States Dollar) *for the purchase of medications* [the medicines] *are consumed very quickly* [and can't last the whole year] *some medicines are used more than the others”*

**Veterinarian 4**

“[the budget for our clinic] *is not sufficient and enough* [..] t*he budget for medication purchase it is around 100,000 ETB* (1 Ethiopian Birr = 0.019 United States Dollar) *per year. But it can cost millions if we truly try to purchase what cattle owners need. What the government budgets only covers around 20-25% of the need* [the rest] *is covered by the owners' purchase from private markets*.”

**Veterinarian 6**

Clinics and other institutions lacked the necessary capacity to respond to disease outbreaks, such as proper housing for livestock, medical drugs storage facilities, and laboratory equipment and reagents to adequately provide the correct diagnosis.

“*We don't have laboratory infrastructure here* [animal health clinic], *you can easily see the lack of attention by facilities we are in* [there is no funding for building clinics]. *It has been 10 years since it was opened to all the ten districts* [smallest administrative units] *of the sub-city but there is not even a single permanent clinic in all of them*.”

**Veterinarian 3**

“*As you can see there is no adequate equipment here. we are trying our best to deliver services with the means we get* [..] *of course, that is obvious* [shortage of facilities] *when there are no adequate facilities; you will be limited from providing complete services*.”

**Veterinarian 4**

The personnel complained about the lack of personal protective equipment to protect themselves when attending sick animals and humans which exposed veterinarians and medical doctors to harm particularly to zoonotic diseases:

*“I have to say we are using* [PPE] *in 50% of cases handling due to shortage of budget. Recently there has been no supply and veterinarians take risk attending cases,* [for example] *during dystocia and mass vaccination some of our vets were using gloves by sharing, otherwise, majority of cases are handled only with gown”*

**Veterinarian 1**

Veterinary professionals reported that their colleagues had been exposed to zoonotic diseases due to poor working conditions, including brucellosis and rabies in their day-to-day work routines:

*“This happened here in our clinic this year,* [two veterinarians] *had to take post-exposure vaccine after exposure,* [after they attended cases without protection] *and* [another one] *is still on the treatment”*

**Veterinarian 1**

*“There is one of our staff who was exposed after handling rabid donkey case, during diagnosis he was in contact with the saliva of the infected animal”*.**Veterinarian 2**

### 3.5 Training and staff development

Lack of training was highlighted as a constraint to the implementation of the OH framework as human and animal health professionals were trained in single-discipline thinking rather than encouraged to collaborate through the provision of interdisciplinary training. Veterinary and medical professionals reported feeling a disconnect between their disciplines due to their different training and institutional cultures. Jijiga University was reported as being the only university offering a tailored course focused on training OH professionals in Ethiopia. Interviewees observed that the current approach to training did not equip them with the skills to communicate and collaborate with each other:

*“We attended* [training] *in Debre Zeit* [..] *ten nurses took part in the training* [..] *starting from examining the dog to prescribing medicines* [There are, however, no efforts] *from the government to upgrade us in our education.*”

**Medical doctor 3**

*“Professionals* […] *have* [a] *skill gap* [on] *reporting, communicating and sample taking. Sample taking and diagnostics should be done quickly* [..] *to enable responding”*

**Medical doctor 5**

Interviewees reported that there were challenges associated with the implementation of government policies and regulations, notably, a lack of coherent approach to dealing with public health challenges in a way that could lead to sustainable health outcomes for humans, animals and the environment.

*“This the major problem* [policy implementation] *in this country, policy and regulation on disease control and prevention is not implemented due to unknown reason. Zoonotic diseases survey is low* [due to low emphasis by government authorities]. *NAHDIC has enough facilities to do One Health work* […]. *This can be corrected if the human health and veterinary sectors collaborated and were more integrated”*

**Researcher 1 AHI**

Additionally, there were calls for improved disease surveillance, for example, reporting of animal diseases and deaths by farmers to the veterinary department:

“*They should report to us* [animal deaths] *like they do when their cattle get ill*”

**Veterinarian 4**

“*It is not like we don't have any contact at all* [the chain of command in exchanging information] *is set in a way that a health focal person at District level has to contact the animal health personnel, rather than we at health centre communicating directly with the district level animal health officers.*

**Medical doctor 4**

### 3.6 The Rabies network in Addis Ababa: An example of One Health approach in public health

Rabies is a prevalent endemic zoonosis that affects a lot of people in Ethiopia. Addis Ababa city has an elaborate rabies response network that is tasked with dealing with rabies cases comprising the public health department, human health clinics, veterinarians, human health personnel, laboratories and community working in the various woredas (districts) in the city.

The stray dog population was described as having increased due to a stop on culling events and poor waste management on dump sites by the municipal authorities which provided a source of food and breeding spaces for stray dogs.

“*Stray dogs are everywhere. They have to be vaccinated or be killed* [..] *I think it is because strychnine has been banned* […]. *They* [veterinarians] *are not doing the vaccination work well* [..] *If there are no stray dogs and dog owners kept their dogs at homes, and got them vaccinated* [it is possible to eradicate rabies]”

**Medical doctor 2**

Interviewees reported high number of free roaming (dogs that belonged to homestead but were allowed to freely roam outside) and stray dogs and a lack of vaccination of domestic dogs which caused the high numbers of rabies cases:

“*There are many cases of rabies.* [We get] *around 15 to 20 cases* [Rabies case every week] *since our health centre is the only vaccine provider* [post-exposure treatment] *for the whole sub-city*”

**Medical doctor 1**

Medical professionals took an elaborate approach to handling rabies cases. In every district, medical professionals were expected to train peers working in smaller district health facilities. Veterinarians were reported as being tasked with vaccinating dogs both in homesteads and stray dogs as a way of controlling the incidence of rabies in Addis Ababa city. The community was also taught how to handle rabies bites before proceeding to the hospital.

“*For rabies, we have jointly done* [canine] *vaccination campaign in Hawassa, Adama, Hossiana, and Arbaminch* [..] *in the future, we have planned vaccination in Jimma, Benishangul and Dire-Dawa* […] *one of the control strategies is awareness creation.*”

**Researcher, EWCA**

Compliance with post-bite measures was, however, low:

*“The public is not aware of the primary measure they should take immediately after a dog bite which is to wash the wound with water and soap* [..] *It has an impact before giving the vaccine*.”

**Medical doctor 7**

Medical professionals were required to inform veterinarians and the community animal health workers to follow up cases of dog bite after a patient was treated. Veterinarians were supposed to track down the dog and have it tied for ten days for rabies observation, as there are no available and affordable laboratories to test dogs for rabies in the area.

“[The hospital focal person] *contact with them* [the veterinary people] *The place of the dog should be accessed. The place where the dog attacked, for the dog not to attack again. Veterinarians' phone numbers are posted here, so we contact them”*

**Medical doctor 4**

“*We gather information about the incidence rates in a different part of the sub-city and the details such as the colour of biting dog and other details. We give this information to them* [veterinary department] *so that they can assess the area and do a control work, especially on dog's incubation period*.”

**Medical doctor 1**

Interviewees explained that there was a dedicated team of veterinarians in each Woreda (district) and an animal health clinic which responded to cases of reported dog bites, mainly to catch and cull the rabid dog. The mobile phone numbers of veterinarians who responded to rabies cases were displayed at the animal health clinics and were also shared with community members via printed materials:

*“We used to give pamphlets,* [we] *have to educate people about the importance of vaccination,* [and we] *need to have vaccines in our stores”*

**Veterinarian 1**

Addis Ababa city administration was described by interviewees as having an elaborate system for reporting and responding to rabies cases. Every dog bite case was recorded by either the veterinary or medical health centres and reported to the Public Health department and the Ministry of Agriculture. The formal reporting channel was however, slow and bureaucratic, with the number of cases aggregated and reported on a weekly or monthly basis only. Follow-up of reported cases was slow or no-existent.

“*We do have a weekly case report to them* […] *the surveillance team will report to sub-city* [public health] *office* [of rabies cases]”

**Medical doctor 6**

There was a lack of coordination and communication from a central point which hampered the efforts to eliminate rabies and constrained collaboration between veterinary and human health departments.

“*We don't have* [intersectoral links and communications] *and that adds the burden on us*”.

**Medical doctor 2**

Understaffing was reported as common in both the human and veterinary health departments. Medical professionals reported that rotations and transfers often led to a low number of staff in health centres and undermined the efficient provision of health services:

“*Generally*, [there is a shortage of professionals in our health centre] t*he manpower is low* [..] *there is even a staff shortage below the recommended standard*”

**Medical doctor 5**

Training of human health professionals on rabies was viewed as having the potential to foster a culture of collaboration among the different sectors' professionals. There was a shortage of professionals working in human health and trained in rabies management; animal health clinics equipped to vaccinate all the dogs in their areas and promptly respond to dog bite cases.

“*We contact one of the trainers from EPHI who gave her contacts in case we face any challenges. We told her what we have encountered and her response was to make the call frequently.* [..] *that we can make the collaboration work*”

**Medical doctor 1**

There was consensus that continuous training of staff could help improve the delivery of health services and increase the number of people working at the intersection of human, animal and environment health, and enhance Ethiopia's capacity to deal with zoonoses:

“*If we have put what we have from the training and work in collaboration, we could have at least been aware of the community of the disease very well. But there is a collaboration problem with the sub-city. No one takes initiative*”

**Medical doctor 2**

There was low funding for animal and human health services. Moreover, there was a lack of sharing of finances due to low and unequal budget allocations. Limited availability of financial resources made it difficult to establish collaboration networks between animal, human, laboratory and environmental health professionals. Human health-focused departments had significantly higher financial resources at their discretion for disease control activities. Low funding for animal health services also constrained the response to dog bites by limiting the capacity for transport and medication availability:

*“Previously, there was a medication shortage. We get medications from EPHI and now we are getting in enough quantities. Other supplies like soap are also available*”

**Medical doctor 1**

*“We get vaccines from the government and they are not available in sufficient quantities as well as in the time that we need them to use”*

**Veterinarian 4**

Interviewees, however, noted that there was an ongoing USAID-funded project that was looking to integrate human health and animal health in the rabies response. The project had built a clinic that operated on OH health principles and was expected to offer vaccination services to dogs and treatment for dog bites. Additionally, the facility was expected to offer counselling services to the victims of dog bites and their families. Interviewees reported that the facility constituted an example of how complex public health services could be tackled through a collaborative approach. The rabies project was regarded as demonstrating the benefits of integrating human and animal health through the OH framework which enabled communication and facilitated collaboration between human and animal health practitioners. The approach was also viewed as facilitating sharing of laboratory expertise and equipment. However, the approach was cited as requiring further support from the Ethiopian government to work with partners and ensure funding and staffing to allow the scaling up of the approach, implemented solely in Addis Ababa, across the country. There was consensus that implementation of OH was necessary to address the challenge of zoonoses in Ethiopia and that OH policy implementation related to reducing rabies cases hinged not only on allocating resources and formal structures to support the scaling of health services but also ensuring community buy-in:

*“We have tried to create awareness about that through health extension workers though nothing has much changed [..]* We are still trying to create awareness when they [community] come here*”*

**Medical doctor 5**

“*The big thing we have observed is the radio and local media can be a newsletter and a different way of communication* [..] *diseases prevention is more of awareness creation*”.

**Researcher, EWCA**

## 4 Discussion

This study explored the current state of One Health (OH) implementation in Ethiopia. The results of the study show that although Ethiopia has developed an OH framework, there has been a slow and incomplete implementation of the framework. The risk of zoonoses across the community was evident as shown by the reports of rabies and other animal diseases by veterinarians and medical professionals. Animal and human health systems continue to function independently with no formal coordination, particularly during disease outbreaks when cross-sectoral collaboration and action to prevent and/or control is necessary. There is a need for a multisectoral OH approach to addressing public health issues at the intersections of human, animal and ecosystem health in Ethiopia, as noted in earlier studies [2,19,21,22].

The Ethiopian government has, in recent decades, increasingly invested in strengthening the animal and human health systems [21]. However, the results of this study revealed that there was an absence of formal and legal linkages between the environmental, veterinary and human health departments. The finding is in agreement with Onyango et al. [21], who reported the lack of formal structures or policies for collaboration between veterinary, human health, wildlife and agriculture departments in Ethiopia. Operationalising OH requires a cross-disciplinary local, national, and global collaborative effort of researchers and policymakers working to attain optimal health for people, animals and the environment [18,19].

The current public health “silo approach” in countries such as Ethiopia often leads to faulty assumptions or generalizations that fail to address the real-world zoonoses challenges faced by their societies [5,20,21]. There is a need for a cultural change within agencies to foster attitudinal change and relationships characterised by collaboration between health care professionals (veterinarians, doctors, extension workers, biologists and workers in environment and natural resources). Moreover, there is a need for changing the institutional culture that continues to separate human, animal and environmental health despite the knowledge that a holistic transdisciplinary approach is needed to respond to complex health problems [19,24].

The results of this study suggest that human and animal health sectors may be constrained in their responses to zoonoses due to limited coordination and bureaucratic process within public health departments. Endemic zoonoses are concentrated in poorer parts of the world, where health and veterinary services are inadequate, and the toll of such diseases is undiagnosed and hidden from view [5]. There is a need for adding the number of prioritised diseases and properly funding their research and control to address the health risks faced by the society. Previous research has recommended that zoonoses can be better addressed through a collaborative multisectoral approach that brings together practitioners in human, animal and environmental health [5,22].

Although there was regular reporting of diseases by doctors and veterinarians to the government and its agencies, communication was inefficient and bureaucratic. Previous studies by Onyango et al. [21] and Pieracci et al. [22] have shown that national human health and veterinary services use different disease reporting procedures and communication channels which makes it difficult to communicate and collaborate. The results of this study show that public health organisations monitor and generate information on human or domestic animal diseases but not both in a manner that is actionable. Research has highlighted the disconnection between veterinary and medical professions as a major constraint to the implementation of the OH framework [2,21].

Previous research has shown that investment in public health and veterinary laboratories, the establishment of joint outbreak and surveillance activities, and intersectoral linkages to tackle zoonotic diseases can ensure that Ethiopia and other countries are prepared to effectively address newly emerging zoonotic diseases. [22]. Lack of public health infrastructure is a common problem for countries in sub-Saharan Africa [10]. Investment in OH approaches that integrates human and animal health service delivery could help Ethiopia improve its health care system by increasing vaccine coverage and improving access to human and animal health services [21,22]. The findings of this study underscore the imperative to invest in and improve the capacities of the environment, animal and human health department to deal with zoonotic disease outbreaks, for example, by increasing the availability of vehicles, personnel, and the cold chain for the maintenance of vaccines and medicines [10].

The results of this study related to the rabies network in Addis Ababa show the benefits that arise from collaboration and synergism across human health, environment and veterinary sectors. Collaborative approaches between disciplines and sectors, as advocated under OH, can provide a roadmap for dealing with wicked problems such as endemic and emerging zoonoses, particularly in the current era of financial, economic, social, environmental and health crises [24]. There is an opportunity for scaling up the OH approach as more success stories become available and provide a demonstration of effective collaboration between key stakeholders [16]. The OH approach is increasingly recognised as a valuable approach to tackling challenges resulting from a result of the interaction of humans, animals, and the environment [3,18]. It can provide a pathway to preventing and responding to emerging threats, particularly those of zoonotic origin [16]. The use of an OH approach provides a nuanced approach that can inform prevention and control measures for emerging infectious diseases than traditional epidemiological approaches [10,16].

The rabies network in Addis Ababa provides a model for other regions of Ethiopia of how OH can be operationalised. This study exposes that although Ethiopia has institutionalized OH at the national level, its implementation at the subnational level has been limited. This is due to a lack of funding, competing priorities, and insufficient coordination platforms [10,21]. The results of this study suggest there are opportunities to harness the existing structure of public service delivery and the establishment of a formal coordination mechanism to implement OH activities, as also suggested by Griffith et al. [10] and Onyango et al. [21].

Food safety is significantly influenced by animal and environmental health [19,24]. Given that demand for animal-source foods such as meat and milk is expected to increase, a OH approach can play an important role in combating zoonoses and antibiotic resistance risks. Improving food safety in today's complex world requires a concerted effort by all sectors; government, consumer organisations and industry must work together to ensure access to safe and quality food [21,23]. There is a need for a OH focus at the human-livestock-wildlife interface to improve food production practices, and ecosystem management and prevent microbial and chemical contamination challenges associated with food safety [23,24]. A OH approach facilitates the implementation of food security and safety by ensuring international food safety standards and World Organization for Animal Health (WOAH) protocols for livestock products are upheld, thus contributing to the control of zoonotic and infectious diseases of animal origin [21]. There is a need to enable farmers to adopt and comply with food quality standards through improved access to resources, information and tailoring food standards to fit the local socioeconomic contexts [24].

Low staffing levels in the Veterinary clinics revealed in the results could affect the delivery of services such as rabies treatment and provision of animal health services. This suggests a need to invest in trained veterinary and public health professionals, as also reported by Hassan et al. [11]. Inaccessibility of public health centres and lack of trained manpower in health services are major constraints to the implementation of a OH approach [2]. There is a need to retain trained veterinary and public health professionals to avoid high turnover associated with poor working conditions [24].

There is a need for community engagement in the design, policy design and implementation. Community involvement can contribute to the reduction of zoonotic diseases at the interface between animal-human and their ecosystem [2,11]. Although there is a consensus among international organisations, government authorities, and academic institutions that the OH concept should be a part of a local community's response to zoonotic infection, the OH concept is rarely implemented at the community level [11,19]. Providing information on zoonotic diseases among communities can reduce exposure to pathogens as the population becomes aware of risky practices [11,19].

One limitation of this study is the small sample size of the participants. We used purposive, snowball and convenience sampling which may have an inherent bias as we aimed to reach OH practising professionals [26]. However, previous research has shown that seventeen participants are enough to attain data saturation [8]. Data saturation is achieved when the ability to obtain additional new information has been attained and when further coding is no longer feasible [9]. Additionally, the topics covered may have been sensitive, and although we assured the respondents of confidentiality, some of the participants could have avoided giving information that would negatively reflect on their organisations.

### 4.1 Policy implications

This study contributes to the literature on OH implementation in Ethiopia and indicates that implementation of OH has, to date, been slow due to a number of issues such as institutional culture, underfunding, and lack of funding and investment in health systems [19,21,22]. The results relating to the Addis Ababa rabies network provide examples of how OH can be scaled up, and highlight the challenges that need to be overcome to realise the full potential of adopting a OH approach to managing zoonoses. This study highlights the imperative for funding and investing at the nexus of human, animal and environmental health systems given the societal impacts of emerging health crises require collaborative and concerted response efforts [4,22]. There is a need to invest in training and retaining staff to increase the number of frontline workers in research and health sector to ensure optimal health for the population. The results confirm the need to involve the community in the implementation of OH as it can reduce disease levels through increased access to information and increased awareness of risk factors [2,10]. Finally, there is a need to change the institutional culture to foster collaborative working and sharing of expertise and resources that can generate more benefits for every resource invested in health [10].

## 5 Conclusions

This study provides an overview of the implementation of the OH framework in Ethiopia. There are gaps in the implementation of the OH framework which constrains the provision of improved human, animal and environmental health in Ethiopia. Moreover, there is a need to involve the community to ensure the success of the OH implementation by getting their buy-in and participation as stakeholders in health management. There is a need to fund and entrench a working health approach working culture across government health institutions to enable sharing of expertise and resources and generate better value for the limited financial resources provided by the government. Finally, the rabies network in Addis Ababa city provides an example of how OH can be implemented and that can be used as a model to scale up OH projects across Ethiopia.

## Declaration of Competing Interest

The authors would like to state that there was no conflict of interest resulting from funding or otherwise.

## Data Availability

Data will be made available on request.
